# Editor's Note

**DOI:** 10.1179/107902611X12968323752951

**Published:** 2011-01

**Authors:** Donald R. Bodner

2011 brings change to the *The Journal of Spinal Cord Medicine* — its new design reflects a new direction, new partnerships, and new opportunities. The journal's new cover identifies *JSCM* as a publication of Maney Publishing and formalizes its status as the official journal of both the Academy of Spinal Cord Injury Professionals and the American Spinal Injury Association (ASIA).

In its 34th year, *JSCM* continues to broaden its influence under the auspices of Maney Publishing. Maney publishes more than 90 academic journals in a variety of disciplines, including science and medicine, and has offices in the UK and the US. Maney's capabilities enable the journal to offer more services to readers and authors. All issues will now be published in print and also online via IngentaConnect. Authors will benefit from Fast Track publication and CrossRef services. Table of contents alerts and RSS feeds will inform readers when new content is available. Maney's subscription services will ensure global availability of *JSCM* through libraries and subscription services.

*JSCM* is now published six times a year, bringing readers issues more frequently and providing more editorial pages for multidisciplinary articles in the field of spinal cord medicine, research and rehabilitation. Addressing the scope of topics in our field requires a broad range of contributions from researchers, scientists, and clinicians. For all prospective authors, the journal's submission process remains the same; papers should be submitted through *JSCM*’'s Editorial Manager site at www.editorialmanager.com/jscm. The Academy of SCI Professionals retains the copyright for all published materials. Beginning with this first issue of 2011, *JSCM* carries the logo of ASIA, which has identified *JSCM* as its official journal.

All of our readers are encouraged to explore www.maneyusa.com to learn more about *JSCM*’'s new publisher and its comprehensive services for authors, readers and subscribers.

Many of our readers are aware of other changes affecting the Academy of SCI Professionals. The Academy, now independent from Paralyzed Veterans of America, has announced a new date and venue for its 2011 annual conference – September 5-7 at the Rio All-Suites Hotel in Las Vegas, Nevada. Abstracts may be submitted until March 14 at the new website www.academyscipro.org.

This spring, ASIA holds its annual scientific meeting from June 4-8, 2011 at the Grand Hyatt Washington Hotel in Washington, DC. Attending these conferences helps all of us to advance the care we render and the research we perform.

For more than 3 decades, this journal has reflected our efforts to improve the lives of people with spinal cord injury. With your support, *JSCM* will continue to thrive as the premier journal in our field.

